# Crosstalk between O-GlcNAcylation and ubiquitination: a novel strategy for overcoming cancer therapeutic resistance

**DOI:** 10.1186/s40164-024-00569-5

**Published:** 2024-11-01

**Authors:** Kai Sun, Yuan Zhi, Wenhao Ren, Shaoming Li, Jingjing Zheng, Ling Gao, Keqian Zhi

**Affiliations:** 1 Department of Oral and Maxillofacial Reconstruction, The Affiliated Hospital of Qingdao University, 1677 Wutaishan Road, Huangdao Distract, Qingdao, 266003 Shandong China; 2 School of Stomatology, Qingdao University, Qingdao, 266003 China; 3 Key Lab of Oral Clinical Medicine, The Affiliated Hospital of Qingdao University, Qingdao, 266003 China; 4 Department of Endodontics, the Affiliated Hospital of Qingdao University, Qingdao, 266003 China; 5 Department of Oral and Maxillofacial Surgery, The Affiliated Hospital of Qingdao University, 1677 Wutaishan Road, Huangdao Distract, Qingdao, 266003 Shandong China

**Keywords:** O-GlcNAcylation, Ubiquitination, Interaction, Cancer, Therapeutic resistance

## Abstract

Developing resistance to cancer treatments is a major challenge, often leading to disease recurrence and metastasis. Understanding the underlying mechanisms of therapeutic resistance is critical for developing effective strategies. O-GlcNAcylation, a post-translational modification that adds GlcNAc from the donor UDP-GlcNAc to serine and threonine residues of proteins, plays a crucial role in regulating protein function and cellular signaling, which are frequently dysregulated in cancer. Similarly, ubiquitination, which involves the attachment of ubiquitin to to proteins, is crucial for protein degradation, cell cycle control, and DNA repair. The interplay between O-GlcNAcylation and ubiquitination is associated with cancer progression and resistance to treatment. This review discusses recent discoveries regarding the roles of O-GlcNAcylation and ubiquitination in cancer resistance, their interactions, and potential mechanisms. It also explores how targeting these pathways may provide new opportunities to overcome cancer treatment resistance in cancer, offering fresh insights and directions for research and therapeutic development.

## Introduction

Cancer development is associated with genetic mutations and influenced by genetic and environmental factors [1]. Treatments options include surgery, radiotherapy, targeted therapy, immunotherapy, gene therapy and cell therapy [2–4].However, challenges such as drug resistance, heterogeneity, and metastasis remain unresolved [5, 6]. In the future, the focus should be on the in-depth exploration of molecular pathogenesis and the development of new strategies such as precision medicine, advanced immunotherapy, and gene editing, with the aiming for personalized treatment.

**Fig. 1:**
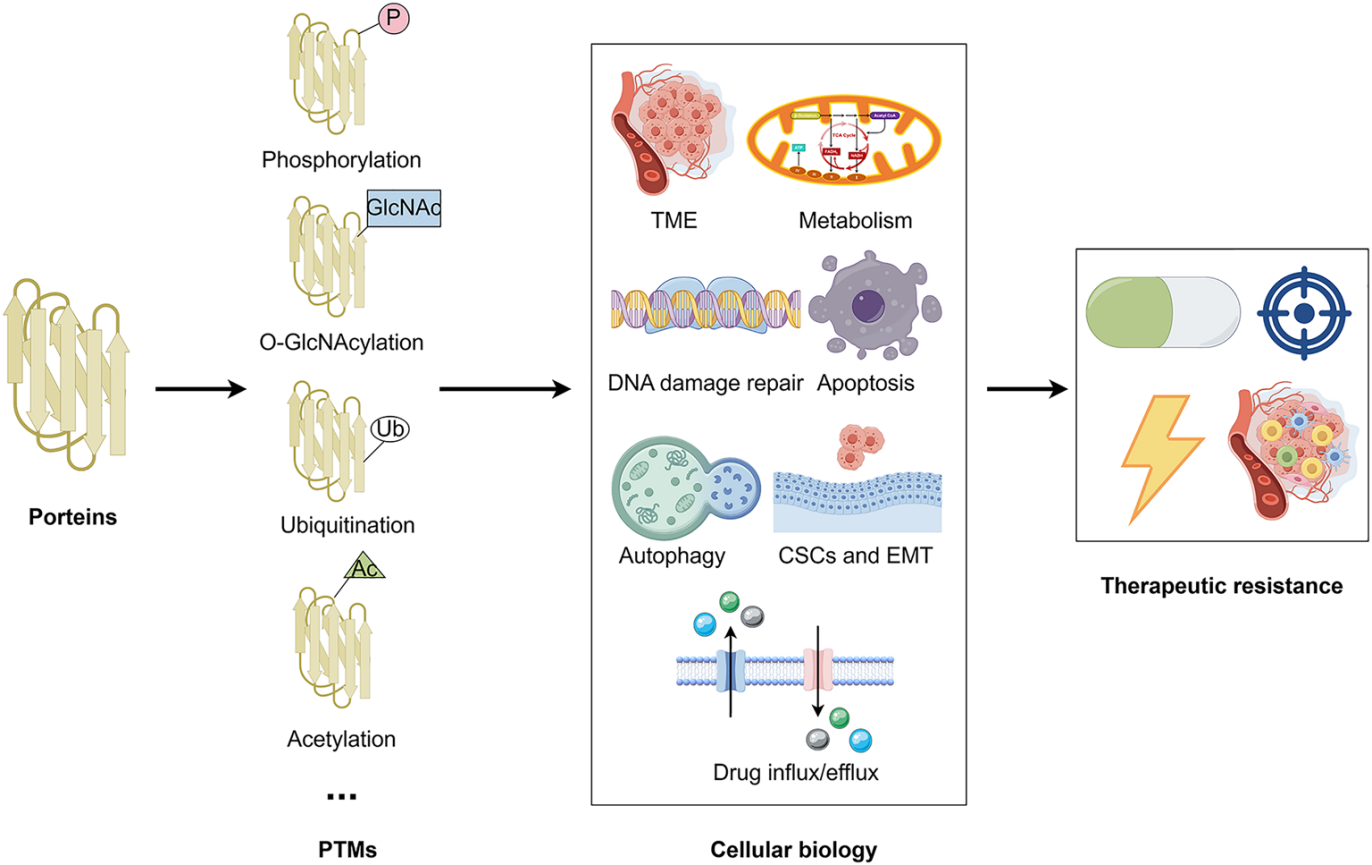
The mechanisms of therapeutic resistance. Increasing drug efflux and decreasing drug influx; Enhancing autophagy; Blocking apoptosis; Inhibiting DNA damage repair; Disordered TME; Promoting CSCs and EMT; Altering energy metabolism and signaling pathway

Therapeutic resistance is a major obstacle to cancer treatment because it enables cancer cells to withstand the effects of chemotherapeutic agents, radiation, and other stimuli [7]. Post-translational modification (PTM) plays a crucial role in this process, impacting the efficacy of chemotherapy by altering the function and stability of the drug's target proteins [8]. For instance, abnormal phosphorylation can activate cell survival pathways, while modifications such as acetylation and methylation can alter the activity of target proteins and affect cellular sensitivity to chemotherapy [9]. These modifications can also disrupt critical biological processes, such as the cell cycle, DNA repair, and apoptosis, thereby contributing to therapeutic resistance (Fig. 1) [10, 11]. Therefore, understanding the role of PTM in therapeutic resistance and developing new therapeutic strategies that target these modifications is essential for improving cancer treatments.

O-GlcNAcylation is a PTM that occurs at serine or threonine residues of intracellular proteins and is regulated by two enzymes: O-GlcNAc transferase (OGT) and O-GlcNAcase (OGA) [12]. O-GlcNAcylation is involved in various biological processes, such as protein function, stability, and cell signalling [13]. In cancer therapy, abnormal O-GlcNAcylation is closely associated with cancer initiation and progression. Recent studies have highlighted its role in chemoresistance. For example, Lee et al*.* found that promoting DR4 O-GlcNAcylation intentionally using 2-deoxy-d-glucose or a high concentration of glucose sensitized those resistant cancer cells to TNF-related apoptosis-inducing ligand (TRAIL). It can be seen that O-GlcNAcylation can enhance the efficacy of chemotherapeutics by increasing the expression of drug pumps in cancer cells and the intracellular concentration of chemotherapeutics [14]. Conversely, Zhang et al*.* demonstrated that O-GlcNAcylation can reduce the efficacy of chemotherapeutics by altering the function and stability of drug target proteins [15]. Investigating the mechanisms of O-GlcNAcylation in chemotherapy resistance and developing intervention strategies that target O-GlcNAcylation is crucial for enhancing the therapeutic outcomes in cancer.

Ubiquitination is a crucial PTM that marks proteins for degradation by attaching ubiquitin molecules to the target protein [16]. In addition to marking proteins for degradation, ubiquitination also plays important roles in protein localization, function, protein kinase activity and interactions with other proteins, thus plays a role in signal transduction [17]. This process requires the combined efforts of several enzymes, including ubiquitin-activating enzyme (E1), ubiquitin-conjugating enzyme (E2), and ubiquitin ligase (E3) [18]. Ubiquitination is vital for maintaining intracellular protein homeostasis and regulating various biological processes [19]. It affects the resistance of tumor cells to chemotherapeutic agents through multiple pathways such as degradation of target proteins, cell cycle regulation, DNA damage repair, signal transduction pathways, and protein interaction networks [20–22]. Moreover, Chu et al*.* reported that O-GlcNAcylation and ubiquitination jointly regulate the proliferation of cancer cells by mutually regulating the stability and function of target proteins [23], offering new insights into cancer therapy resistance. However, reviews on the interplay between O-GlcNAcylation and ubiquitination in therapy resistance are scarce.

This review comprehensively summarizes the current understanding and potential mechanisms of O-GlcNAcylation and ubiquitination in cancer therapy resistance, highlighting their role in regulating therapy resistance and suggesting that targeting O-GlcNAcylation and ubiquitination could help overcome therapeutic resistance.

## O-GlcNAcylation and ubiquitination

### Important O-GlcNAcylation regulators

O-GlcNAcylation is a protein PTM that occurs on serine or threonine residues of intracellular proteins and is regulated by the enzymes OGT and OGA. It plays a role in several biological processes, including protein function, stability, and cell signaling. Abnormal O-GlcNAcylation has been implicated in several diseases, including diabetes, neurodegenerative diseases, and cancer [24–26].

OGT, a crucial enzyme in the O-GlcNAcylation process, attaches GlcNAc from uridine diphosphate N-acetylglucosamine (UDP-GlcNAc) as the donor substrate to serine or threonine residues on proteins. It exists in three different splice forms: ncOGT, mOGT, and sOGT. sOGT and ncOGT are primarily found in the nucleus and cytoplasm, whereas EOGTis located in the endoplasmic reticulum and mOGT is located in the mitochondria [27]. In cancer, OGT facilitates tumor cell growth and survival, enhances invasion and metastasis, modulates the tumor microenvironment (TME), contributes to chemoresistance, and affects gene expression and epigenetics [28–30]. For instance, OGT enhances the PI3K/AKT/mTOR signaling pathway by modifying fatty acid synthase (FASN), which promotes hepatoma cell proliferation [31]. Moreover, it influencd transcriptional activation of connective tissue growth factor and Snail family transcriptional repressor 1 (SNAIL) by regulating the O-GlcNAcylation of CW-like zinc finger 2 of the MORC family (MORC2). That affected breast cancer (BC) cell migration and invasion [32]. Additionally, OGT plays a critical role in chemoresistance in hepatocellular carcinoma (HCC) and other cancers [33].

In contrast to OGT, OGA, which removes the Ser/Thr-O-GlcNAc from proteins during the O-GlcNAcylation process, is encoded by the meningioma-expressed antigen-5 (MGEA5) gene and has two splice forms: sOGA and ncOGA. ncOGA is located in both the cytoplasm and nucleus and contains an N-terminal O-GlcNAc hydrolase domain and a C-terminal histone acetyltransferase-like domain. sOGA, containing only the hydrolase domains, is distributed in the endoplasmic reticulum and lipid droplets [27, 34]. In pancreatic ductal adenocarcinoma (PDAC) cells, OGA influences the regulation of NF-κB p65 subunit and the O-GlcNAcylation of IKα and IKβ, leading to the phosphorylation of p65 Ser-536 and decreased NF-κB transcriptional activity, thus promoting cancer growth [35]. Furthermore, OGA plays a significant role in tumor dynamics by regulating protein stability, gene expression, chemotherapy resistance, and TME [36, 37].

**Fig. 2:**
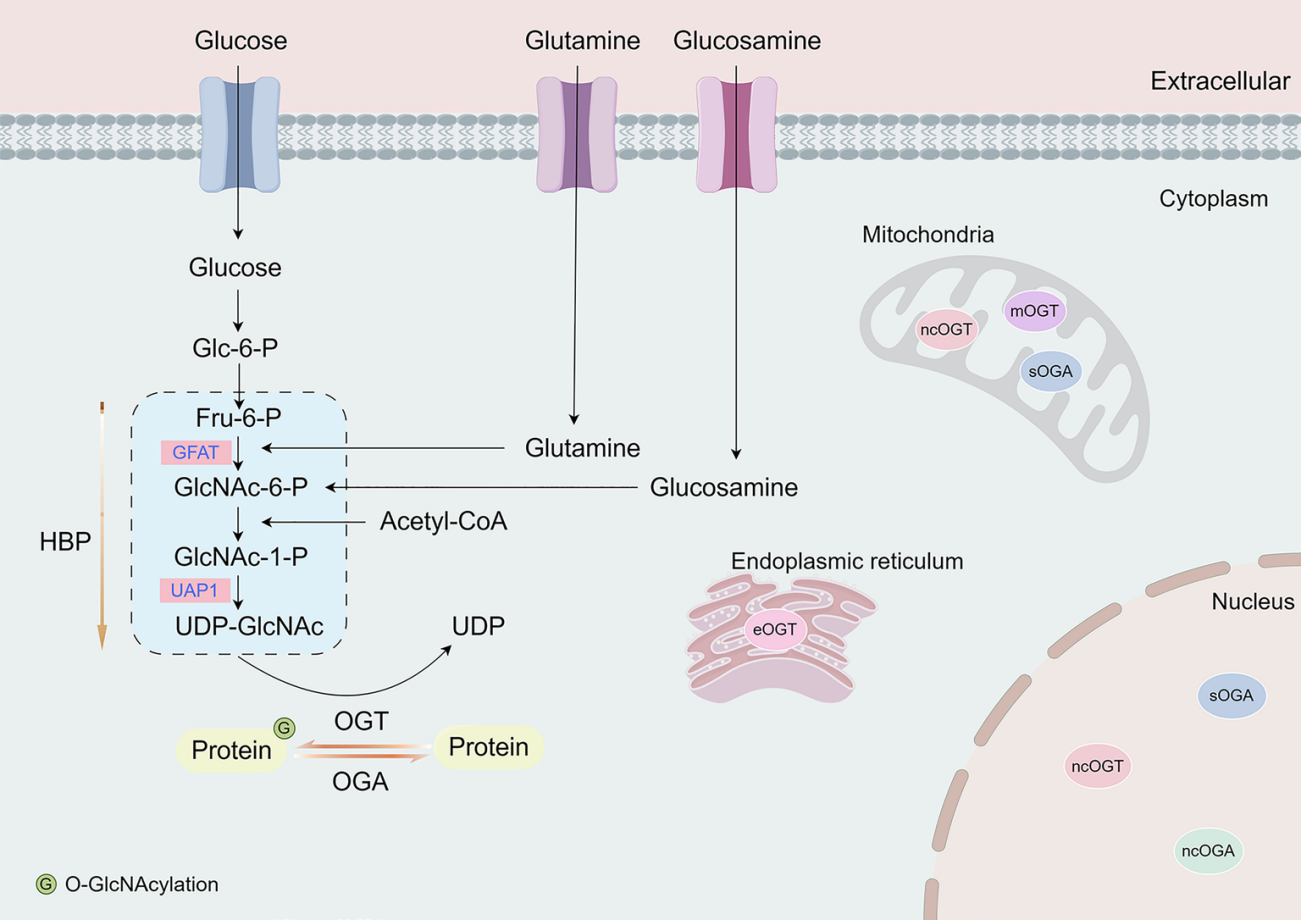
O-GlcNAcylation cycling and UDP-GlcNAc synthesis. The dynamic process of the O-GlcNAcylation cycle is controlled by OGT and OGA by adding and removing UDP-GlcNAc to proteins. Specifically, OGT utilizes UDP-GlcNAc to generate proteins, whereas OGA reverses this process by deglycosylation. This regulation of O-GlcNAcylation plays a key role in the control of various cellular activities and operations. Proteins labeled G have been shown to be O-GlcNAcylation. The hexosamine biosynthetic pathway (HBP) is an auxiliary pathway produced by glycolysis. Glucose is converted to glucosamine-6-phosphate (GlcN-6-P) by glutamine fructose-6-phosphate transaminase (GFAT1). Subsequent reactions produce the substrate of O-GlcNAc, UDP-GlcNAc. OGT has several isoforms in different cellular compartments: nucleoplasmic OGT (ncOGT) and truncated short OGT (sOGT) are found predominantly in the nucleus and cytoplasm; mitochondrial OGT (mOGT) and ncOGT operate within the mitochondria. The EGF structural domain-specific O-GlcNAc transferase (EOGT) is mainly located in the endoplasmic reticulum (ER) and mediates the transfer of UDP-GlcNAc to extracellular proteins. There are two main isoforms of OGA: the nucleocytoplasmic long isoform (lOGA); and the short isoform of OGA present in mitochondria (sOGA)

UDP-GlcNAc is a crucial substrate for O-GlcNAcylation, which plays a role in many biological processes such as cell signaling, gene expression regulation, protein stability, metabolism, and cell cycle regulation [38]. The synthesis of UDP-GlcNAc is mainly controlled by the hexosamine biosynthetic pathway (HBP) [39], which converts 3–5% of glucose into UDP-GlcNAc. This substrate is essential for the production of glycoproteins, glycolipids, proteoglycans, and glycosaminoglycans. The HBP shares its initial steps with glycolysis, followed by a specific synthetic pathway. Key enzymatic steps involve glutamine-fructose-6-phosphate aminotransferase (GFAT/GFPT), which converts fructose-6-phosphate and glutamine into glucosamine-6-phosphate and glutamate, resulting in the UDP-GlcNAc production [40]. HBP is often abberrantly activated in cancer. Increased supply of nutrients such as glucose, glutamine, fatty acids, and amino acids enhances HBP flux and UDP-GlcNAc availability. O-GlcNAcylation, regulated by UDP-GlcNAc substrates, integrates HBP flux with other metabolic pathways and signaling pathways, promoting cancer development (Fig. 2).

### Mutual regulation between O-GlcNAcylation and ubiquitination

Ubiquitination modification is a critical process in protein degradation that covalent attachment of attaching ubiquitin molecules to target proteins. Such modification includes forms such as monoubiquitination, polyubiquitination (primarily through Lys48 connections), chain ubiquitination (e.g., Lys63 connections), branching, and internal modifications [41, 42]. The process of ubiquitination, which includes ubiquitin activation, transmission, and linking, is crucial for maintaining cellular stability and managing biological stress [43]. Ubiquitination affects protein stability and degradation and plays a role in many biological processes such as cell signaling, cell cycle, DNA damage repair, inflammation, immune response, apoptosis, chromatin remodeling, chaperone-mediated folding, endocytosis, and membrane protein turnover [44–46]. Ubiquitination has implications in cancer, neurodegenerative diseases, genetic disorders, autoimmune diseases, and metabolic diseases [45, 47, 48].

Strikingly, O-GlcNAcylation significantly affects ubiquitination functions [49]. The roles of O-GlcNAcylation in ubiquitination primarily focus on four aspects: (1) O-GlcNAcylation can inhibit the ATPase activity of the 26S proteasome. Zhang et al*.* showed that glycosylation modifies the 26S proteasome, inhibiting its ATPase activity and the proteolysis of transcription factor Sp1 and hydrophobic peptides [50]. (2) Reduction of ubiquitin-mediated protein degradation: O-GlcNAcylation and phosphorylation have the same modification site, and there is competition and mutual inhibition in the process of post-translational modification. The O-GlcNAcylation site is often located in the PEST sequence associated with protein degradation (sequence rich in proline, glutamate, serine, and threonine), which is the signal of the target protein targeted in the ubiquitin proteasome pathway [51]. PEST sequence activation usually occurs after phosphorylation. Due to the competitive relationship between O-GlcNAc and phosphorylation, O-GlcNAc modification can be assumed to counteract phosphorylation and thus protect proteins from ubiquitin-mediated protein degradationubiquitin-mediated protein degradation of certain substrate proteins [52]. For instance, Yang et al*.* found that hepatitis B virus (HBV) infection increased O-GlcNAcylation of YTHDF2, inhibiting its ubiquitination and thereby boosting its protein stability and oncogenic potential [53]. Similarly, Hu et al*.* observed that HBP and O-GlcNAcylation enhance the natural antiviral response to HBV by regulating the ubiquitination of SAMHD1, affecting its stability and antiviral activity [54]. (3) Modification of ubiquitination-related enzymes: O-GlcNAcylation modifies enzymes related to ubiquitination and alters their functions. For example, elevated extracellular glucose levels increase the stability of DOT1L protein via HBP. DOT1L is glycosylated by O-GlcNAcylation at an evolutionarily conserved S1511 site at the C-terminus, which influences its interaction with UBE3C, affecting its ubiquitination and subsequent degradation. This modification affects the methylation of H3K79 and the expression of DOT1L target genes such as HOXA9/MEIS1, promoting the proliferation of MLL-fused leukemia cells [55]. Additionally, the S-phase kinase-associated protein 2 (Skp2) F-box protein is the substrate recruiting component of the SCF ((Skp1-Cullin 1-F-box) type of E3 ubiqutin-ligase complexes. The SCF complex consists of four components: the invariable component Skp1, Rbx1 and Cullin1, and the interchangeable F-box protein that functions as a receptor for target proteins [56]. The substrate protein is recognized in the process of protein ubiquitin-mediated protein degradation, thus promoting protein degradation through the ubiquititation-proteasome pathway. Feng et al*.* suggested that the O-GlcNAcylation of SKP2, a significant E3 ubiquitin ligase, enhances its binding with SKP1, improving its ligase activity, crucial for promoting HCC proliferation [57]. (4) Indirect effects on protein ubiquitination: O-GlcNAcylation also indirectly affects protein ubiquitination. For example, OGT-mediated O-GlcNAcylation can activate the ubiquitin-mediated protein degradation of transcription factor FOXA2, reducing the transcription of E-cadherin and promoting the migration and invasion of HCC cells [58]. Conversely, Chen et al*.* discovered that O-GlcNAcylation activates the transcriptional activity of Nrf1, promoting the ubiquitination and degradation of the cap’n’colla basic region-leucine zipper (CNC-bZIP) protein [59]. In summary, O-GlcNAcylation plays a crucial role in regulating the growth, proliferation, and invasion of cancer cells. One of the mechainsms of that is O-GlcNAcylation modulated the ubiquitination of target proteins and then affected their stability and function.

Ubiquitination also influences the level of O-GlcNAcylation in cancer. For instance, Tang et al*.* demonstrated that the ubiquitin-specific peptidase 8 (USP8) stabilized OGT by removing its K48-specific poly-ubiquitination at the K117 site. OGT can O-GlcNAcylate the solute carrier family 7, member 11 (SLC7A11) at Ser26, inhibiting ferroptosis and promoting the progression of HCC [60]. Additionally, Peng et al*.* discovered that OGT is a ubiquitination target of the HECT-type E3 ubiquitin ligase E6AP, leading to the degradation of OGT and reduced O-GlcNAcylation when overexpressed in HEK293 cells [61]. In summary, O-GlcNAcylation significantly affects ubiquitination functions in four aspects, including inhibition of the ATPase activity of the 26S proteasome, reduction of ubiquitin-mediated protein degradation, modification of ubiquitination-related enzymes and indirect effects on protein ubiquitination. Therefore, O-GlcNAcylation not only regulates the biogenesis and function of ubiquitination but is also affected by ubiquitination.

## O-GlcNAcylation involved in regulating cancer therapeutic resistance

**Fig. 3:**
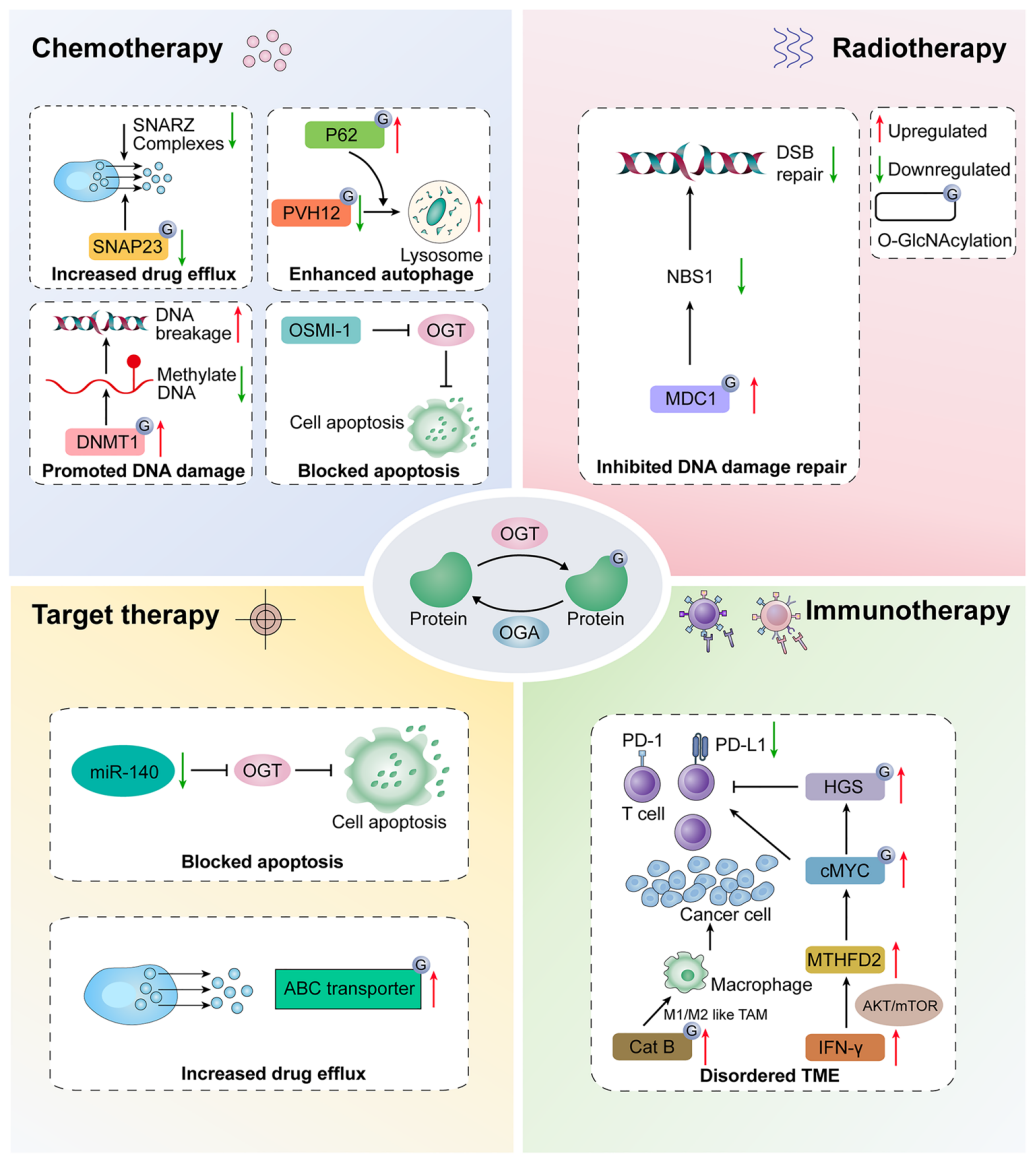
Altered O-GlcNAcylation in cancer therapeutic resistance. O-GlcNAcylation is dynamically modulated by OGT and OGA, and these O-GlcNAcylation proteins play vital roles in therapeutic resistance. (1) In chemotherapy: O-GlcNAcylation can reduce chemosensitivity through facilitating drug efflux, autophagy, DNA damage repair and apoptosis. (2) In radiotherapy: O-GlcNAcylation can promote radioresistance via DNA damage repair. (3) In targeted therapy: O-GlcNAcylation can increase drug efflux and blocking apoptosis, thereby weakening the efficacy of target therapy. (4) In immunotherapy: O-GlcNAcylation can remodel the TME, and further lead to immunotherapy resistance

In recent years, significant advances in cancer treatment strategies have emerged, including immunotherapy, targeted therapy, individualized therapy, and radiotherapy. However, chemotherapy resistance remains a major challenge. O-GlcNAcylation plays a critical role in cancer cell growth, invasion and metastasis. This section primarily focus how O-GlcNAcylation influences cancer response to therapies through various mechanisms (Fig. 3, Table 1).

**Table 1:** Altered O-GlcNAcylation in cancer therapeutic resistance

Therapeutic resistance	Mechanisms	Cancer types	O-GlcNAcylation protein	Functions	Ref
Chemoresistance	Drug transport	Ovarian cancer	SNAP-23	Cisplatin resistance	[62]
	Autophagy	Ovarian cancer	SNAP-29	Cisplatin resistance	[73]
		Lung carcinoma	YAP	Cisplatin resistance	[76]
		Glioblastoma	/	Temozolomide resistance	[99]
	DNA damage repair	HCC	DNMT1	Cisplatin resistance	[84]
		HCC	Polη	Cisplatin resistance	[88]
	Apoptosis	Prostate cancer	OGT expression	Docetaxel resistance	
Radioresistance	DNA damage repair	Breast cancer	MDC1	Radioresistance	[86]
Targeted therapy resistance	Drug transport	Ovarian cancer	ABC transporter	Cisplatin resistance	[66]
Immunotherapy resistance	TME	HNSCC	HGS	Anti-PD-1 resistance	[95]
		Pancreatic cancer	cMYC	Anti-PD-1 resistance	[96]

### O-GlcNAcylation-induced alterations in drug concentration

The study of the intracellular concentration of anticancer drugs is essential to assess their efficacy. Cisplatin, a platinum-based chemotherapeutic agent that approved by the US Food and Drug Administration in 1978, is used to treat testicular, ovarian, and bladder cancer. Resistance to cisplatin can arise through several mechanisms, such as reduced intracellular accumulation of the drug, increased drug detoxification, enhanced DNA repair capacity, tolerance to DNA damage, and increased cellular resistance to death signals [62]. Recent studies suggest that reducing OGT expression lowers O-GlcNAcylation on SNAP-23, facilitating the assembly of SNARE complexes and the production of exosomes in ovarian cancer cells (Fig. 3). This O-GlcNAcylation-dependent mechanism contributes to chemoresistance by promoting the efflux of cisplatin through exosomes [63]. In addition, drug transporters play a crucial role in regulating drug transport into and out of tumor cells. Increased efflux and decreased influx are primary factors in drug resistance [64, 65]. Many anticancer drugs, including cisplatin and paclitaxel, are substrates for ABC transporter family proteins, which are responsible for drug efflux [66, 67]. The ABC transporter family proteins consist of transmembrane domains (TMDs) and an ATP-binding region in the cytoplasm [68], which after binding ATP, transports substrates to the membrane by hydrolyzing ATP. The TMDs form a channel that allows drugs to pass through by binding to the transported substrates [69]. Li et al*.* showed that targeting AKT enhanced the cytotoxic effects of taxol and cisplatin by downregulating ABC transporters [66]. Additionally, studies have shown that hypo-O-GlcNAcylation inhibits AKT activation in HepG2 cells [33].

### O-GlcNAcylation modulates autophagy

Autophagy is a cell survival mechanism that maintains nutrient homeostasis and responds to stress by degrading and recycling intracellular components [70]. In chemotherapy, autophagy can confer resistance to cancer cells by degrading chemotherapeutic agents, removing damaged organelles to reduce oxidative stress and DNA damage, and inhibiting apoptosis signaling pathways [71, 72]. O-GlcNAcylation is involved in autophagy activation and the formation of autophagosomes that process chemotherapy drugs. For example, knocking down of OGT increased cisplatin-induced autophagy, reduced apoptosis, and promoted autophagy lysosome formation due to decreased O-GlcNAcylation of synaptosomal-associated protein 29 (SNAP-29), which, in turn, promoted SNARE complex formation and autophagy flux [73]. In another study, Pellegrini et al*.* discovered that small molecule 15 (SM15) inhibited basal autophagy flux by preventing the fusion of autophagosomes and lysosomes, thereby enhancing apoptosis associated with reactive oxygen species production. SM15 also blocked SNARE complex formation by increasing O-GlcNAcylation of SNAP29, suggesting a key role for O-GlcNAcylation of SNAP29 in impeding autophagy flux and influencing tumor therapy [74]. Additionally, autophagy selectively degrades other intracellular components, including some oncogenes. Yes-associated protein (YAP) acts as a critical transcriptional co-activator that fosters cancer progression by promoting cell growth and enabling cells to evade programmed cell death [75]. The von Hippel-Lindau tumor suppressor protein (pVHL) curbs the proliferation and migration of lung cancer cells by regulating the lysosomal degradation of YAP, thus enhancing the chemotherapy sensitivity to cisplatin. O-GlcNAcylation can inhibit this process, providing new insights into the tumor-suppressive effects of Pvhl (Fig. 3) [76]. Therefore, O-GlcNAcylation-mediated autophagy plays a significant role in chemoresistance.

### Role of O-GlcNAcylation in DNA damage repair

DNA damage is a critical factor in cancer development and a potential target for cancer [77] treatments such as chemotherapy and radiotherapy, which destroy cancer cells by inducing DNA damage. However, cancer cells can activate their DNA repair mechanisms to counteract these treatments, making strategies targeting DNA damage often ineffective [78, 79]. Certain DNA damage repair proteins are crucial in developing resistance to chemoradiotherapy. Additionally, the levels of these proteins are tightly linked to O-GlcNAcylation. For example, DNA methyltransferase 1 (DNMT1), a key enzyme in DNA methylation [80], is associated with resistance to cisplatin in several cancers, including non–small cell lung cancer (NSCLC), lung cancer, and ovarian cancer [81–83]. To explore the role of O-GlcNAcylation in chemotherapy resistance further, Shin et al*.* found that O-GlcNAcylation of DNMT1 at Ser 878 hampers its DNA methylation ability, leading to decreased overall DNA methylation, especially in partially methylated domains. This reduction in methylation correlates with higher DNA damage and increased cell apoptosis (Fig. 3) [84]. DNA double strand breaks (DSBs), one of the most detrimental types of DNA damage, can be repaired by slow but highly accurate homologous recombination repair (HRR) [85]. Averbek et al*.* investigated DSB rejoining, protein accumulation, and chromatin states after treating cells with OGT or OGA inhibitors, focusing on the role of GlcNAcylation in DNA damage response (DDR) and chromatin remodelling [86]. Notably, MDC1's O-GlcNAcylation increases upon irradiation, aiding the recruitment of other HRR factors such as CtIP and BRCA1, which also show increased O-GlcNAcylation following radiotherapy (Fig. 3) [86]. Furthermore, DNA polymerase η (Polη) facilitates translesion DNA synthesis (TLS) across ultraviolet- and cisplatin-induced lesions, linked to skin cancer and chemoresistance [87] development. Notably, O-GlcNAcylation of human Polη at Thr 457 is crucial for TLS regulation and maintaining genome stability. A deficiency in O-GlcNAcylation impairs TLS and heightens cisplatin sensitivity, suggesting a novel approach to enhance chemotherapy effectiveness [88].

### Remodeling of tumor microenvironment by O-GlcNAcylation

The TME consists of a variety of cell types and molecules, such as immune cells, vascular endothelial cells, stromal cells, and the extracellular matrix (ECM). These components significantly influence tumor growth, invasion, metastasis and drug resistance [89, 90]. Recently, the role of O-GlcNAcylation in TME has gained attention, especially in tumor immunotherapy. For instance, in B16 melanoma cells, increased O-GlcNAcylation in the TME has been shown to reduce the production of inflammatory cytokines, thereby promoting cancer progression through suppressing p38 MAPK activity and enhancing the ERK1/2 signaling pathway [91]. Additionally, Shi et al*.* demonstrated that enhanced glucose metabolism could drive cancer metastasis and chemotherapy resistance by increasing O-GlcNAcylation of cathepsin B in tumor-associated macrophages, thus facilitating their maturation and secretion [28]. Recent breakthroughs in cancer immunotherapy, such as pembrolizumab, capitalize on the discovery that many tumors evade T cell attacks by upregulating PD-L1. Pembrolizumab works by inhibiting the PD-1/PD-L1 interaction, rejuvenating T cell activity to target and destroy tumor cells, which shows promising antitumor effects [92–94]. In addition, emerging studies indicate that O-GlcNAcylation of certain proteins, such as the hepatocyte growth factor–regulated tyrosine kinase substrate (HGS), can trigger T cell-mediated antitumor responses by affecting the lysosomal degradation of programmed-death ligand 1 (PD-L1) [95]. Similarly, Shang et al*.* found that the enzyme methylenetetrahydrofolate dehydrogenase 2 promotes the folate cycle and the production of critical metabolites including UDP-GlcNAc. This, in turn, boosts O-GlcNAcylation of proteins such as cMYC, which stabilizes cMYC and promotes the transcription [96] of PD-L1, offering a theoretical basis for immunotherapy of pancreatic cancer (Fig. 3).

### Other mechanisms mediated by O-GlcNAcylation

Furthermore, other mechanisms such as glucose metabolism and abnormal apoptosis also play roles in therapeutic resistance. The influence of O-GlcNAcylation on these processes remains be an active area of research.

Apoptosis, or programmed cell death, is a critical biological process. The B-cell lymphoma 2 (BCL-2) family of proteins plays a central role in regulating this process. In cancer, overexpression of anti-apoptotic proteins of the BCL-2 family can leads to chemoresistance [97]. For example, in Mantle cell lymphoma, the increased production of the anti-apoptotic protein Bcl-xL and decreased levels of the pro-apoptotic protein Bax, combined with decreased OGT activity, contribute to resistance to the drug bortezomib [98]. Additionally, research by Leonel et al*.* has shown that decreased O-GlcNAcylation enhanced the sensitivity of Glioblastoma cells to the chemotherapy drug temozolomide by inhibiting autophagy and promoting cell apoptosis [99]. Furthermore, studies indicate that OSMI-1, either alone or combined with doxorubicin, was effective in decreasing OGT enzyme levels and increasing apoptosis in prostate cancer cells, presenting a potential alternative treatment strategy for enhancing cancer therapy effectiveness [100]. Likewise, treatment with docetaxel upregulates miR-140 expression in prostate cancer cells, and miR-140 can directly bind to the 3′-untranslated region of OGT mRNA to suppress its expression, thereby enhancing the drug sensitivity of PC cells to docetaxel (Fig. 3) [101]. Therefore, OGT is expected to be a potential therapeutic target to improve drug sensitivity by regulating cell death.

Altered energy metabolism is a characteristic of cancer cells. Recent research has demonstrated that cisplatin increases O-GlcNAcylation of proteins by affecting the function of OGT, OGA, and AMPK, both in vitro and in vivo. This alteration a decrease in the reduced activation of AMPK and a decrease in the phosphorylation of glutamine-fructose-6-phosphate aminotransferase 1 (GFAT1), thereby increasing GFAT1 activity and enhancing the production of the substrate UDP-GlcNAc following cisplatin treatment [36]. Nie et al*.* suggested that inhibiting O-GlcNAcylation of phosphoenolpyruvate carboxykinase 1 (PCK1) at threonine 255 reduces colon cancer cell proliferation, decreased glycolysis, incerased tricarboxylic acid cycle activity, and inhibitd tumor growth in xenograft models. Another study found that decreased levels of PCK1 increase O-GlcNAcylation of CHK2 at threonine 378, which destabilizing CHK2 and preventing its dimerization. This disruption leads to enhanced CHK2-mediated phosphorylation of Rb and promotes rapid growth of HCC[102].

## Ubiquitination and cancer therapeutic resistance

**Fig. 4:**
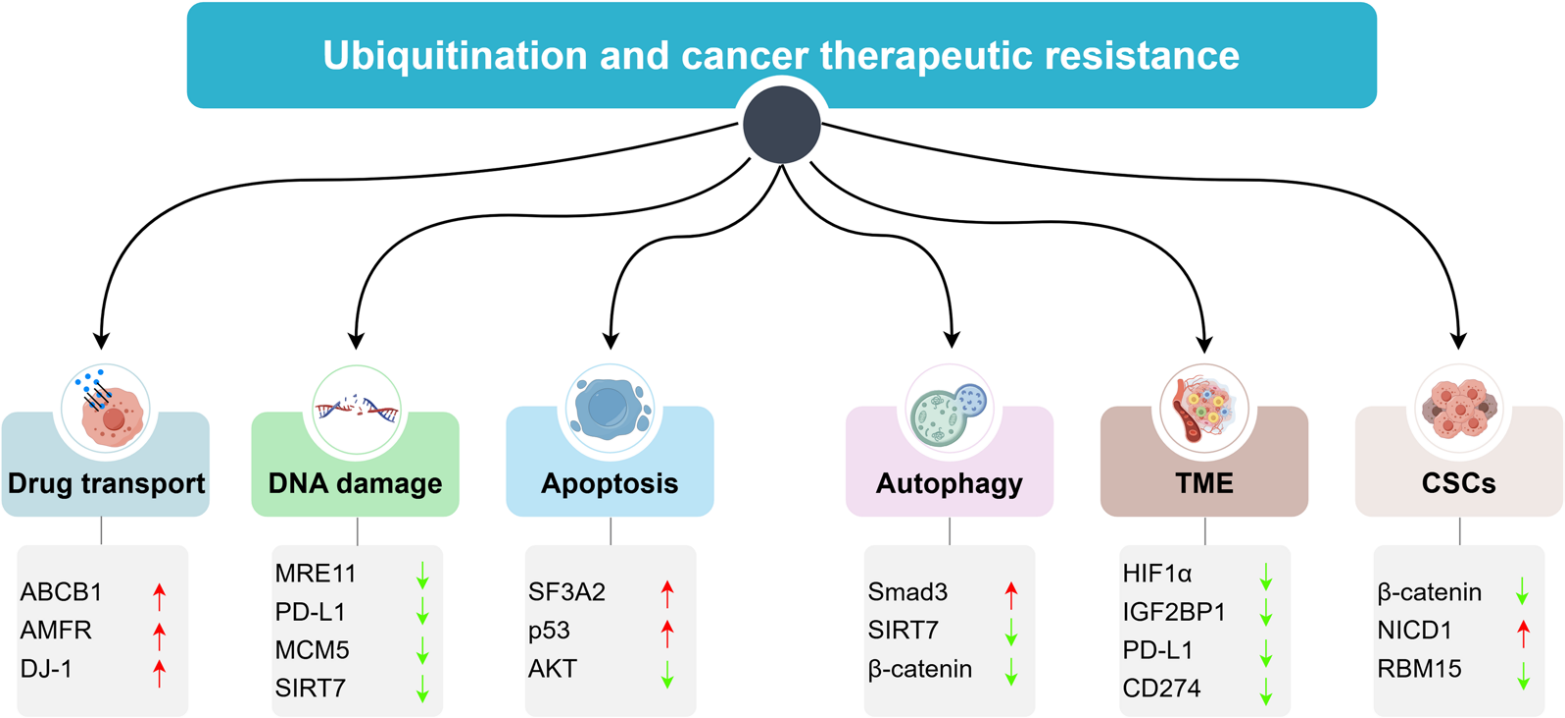
Ubiquitination associated with cancer therapeutic resistance. Ubiquitination can affect sensitivity to cancer therapy through multiple mechanisms, including modulation of drug transport (such as ABCB1, AMFR and DJ-1), DNA damage repair (such as MRE11, PD-L1, MCM5 and SIRT7), apoptosis (such as SF3A2, p53 and AKT), Autophagy (such as Smad3, SIRT7, β-catenin), TME (such as HIF1α, IGF2BP1, PD-L1 and CD274) and CSCs (such as β-catenin, NICD1 and RBM15)

Ubiquitin (Ub) is a 76-amino acid protein that is highly conserved in all eukaryotes. It serves as one of the most prevalent protein post-translational modifiers, regulating various crucial cellular functions [103]. The process of ubiquitination involves the covalent attachment of ubiquitin to a target protein, facilitated by a series of enzymes [104]. This modification enables ubiquitin to regulate the degradation of different protein substrates, thus playing a role in nearly all vital regulatory functions related to life, such as the cell cycle, proliferation, apoptosis, differentiation, gene expression, immunity, and so on [105]. Ubiquitination affects cancer progression through multiple pathways, acting as a double-edged sword that can both promote and inhibit cancer development. Increasing evidence suggests that ubiquitination also impacts cancer therapeutic resistance through several mechanisms, including DNA damage repair, apoptosis, TME, autophagy, and cancer stem cells (CSCs) (Fig. 4, Table 2).

**Table 2:** Ubiquitination and cancer therapeutic resistance

Therapeutic resistance	Mechanisms	Cancer types	Ubiquitination protein	Functions	Ref
Chemoresistance resistance	Autophagy	NSCLC	SIRT7	Cisplatin resistance	[120]
		HCC	SIRT7	Sorafenib resistance	[121]
	DNA damage repair	Breast cancer	SIRT7	Cisplatin resistance	[112]
	Apoptosis	TNBC	SF3A2	Cisplatin resistance	[115]
		Ovarian cancer	p53	Cisplatin resistance	[116]
		Gastric cancer	AKT	MDR	[117]
	CSCs	HCC	DJ-1	Cisplatin resistance	[108]
		LUAD	β-catenin	Osimertinib resistance	[133]
		NSCLC	NICD1	Drug resistance	[135]
		Prostate cancer	RBM15	Docetaxel resistance	[136]
	TME	HCC	HIF1α	Sorafenib resistance	[125]
Radioresistance	DNA damage repair	TNBC	MRE11	Radioresistance	[109]
Immunotherapy resistance	DNA damage repair	HNSCC	PD-L1	Anti-PD-1 resistance	[110]
	TME	HCC	IGF2BP1	Anti-PD-1 resistance	[130]
	Autophagy	PDAC	CD274	immunochemotherapy	[132]

### Ubiquitination and drug transport

Ubiquitination can modulate therapeutic resistance by influencing ABC transporters. For example, Zou et al*.* found that the ABC subfamily B member 1 (ABCB1) interacts directly with the E3 ubiquitin ligase membrane–associated RING-CH 8 (MARCH8). Rutaecarpine can enhance the ubiquitination and degradation of ABCB1 by upregulating MARCH8, thereby counteracting ABCB1-mediated multidrug resistance (MDR) [106]. Similarly, Saeed et al*.* discovered that betulinic acid (BetA) can also reduce MDR by affecting ABCB1 ubiquitination and AMFR activity in treatment-resistant tumors [107]. Additionally, in chemoresistant HCC, the STAT3-mediated ubiquitin-mediated protein degradation of DJ-1 can influence chemotherapy resistance in HCC cells by regulating CSC markers and ABC transporters [108]. However, the role of ubiquitination on the regulation of drug concentration has not been reported yet.

### Ubiquitination and DNA damage repair

Ubiquitination affects the expression of DDR-related proteins and thus affect the effectiveness of cancer treatments. Liu et al*.* observed that the E3 ubiquitin ligase Ring finger protein 126 (RNF126) becomes active after irradiation (IR) therapy. It mediates ubiquitination of meiotic recombination 11 homolog 1 (MRE11) at K480 in triple-negative BC (TNBC) [109]. This ubiquitination enhances MRE11's DNA exonuclease activity, leading to increased RPA binding and ATR phosphorylation. Consequently, this promotes a sustained DDR skewed toward HRR, increasing TNBC sensitivity to radiotherapy [109]. Additionally, the expression of another E3 ligase, FBXO22, correlates with NSCLC cel sensitivity of NSCLC cells to ionizing radiation (IR) and cisplatin. De et al. demonstrated that FBXO22 activates PD-L1 ubiquitination and degradation, thereby increasing NSCLC cells' sensitivity to DNA damage. This may enhance the efficacy of immune checkpoint blockade [110]. Furthermore, in cisplatin-resistant HNSCC, lnc-POP1-1 enhances DNA repair in HNSCC cells by interacting with minichromosome maintenance deficient 5 (MCM5) and slowing its ubiquitin-mediated protein degradation [111]. Moreover, the balance between ubiquitination and deubiquitination is essential for maintaining intracellular homeostasis. Recently, Su et al. showed that high expression of USP17L2 regulates DDR by increasing SIRT7 protein stability, potentially sensitizing cancer cells to chemotherapy in BC [112]. In summary, these findings highlight the role of ubiquitination in modulating DDR and influencing resistance to cancer therapies.

### Ubiquitination and apoptosis

In the fight against cancer, therapies can eliminate some tumor cells by initiating apoptosis [113]. However, when the regulation of apoptosis signaling pathways is disrupted, tumor cells may gain a survival advantage, leading to resistance against therapy [114]. Research has shown that ubiquitination played a crucial role in the apoptosis of tumor cells. For instance, Deng et al. revealed that E3 ubiquitin-protein ligase UBR5 promotes the ubiquitin-dependent degradation of Splicing factor 3a subunit 2 (SF3A2) in TNBC, which leads to cisplatin resistance by affecting both extrinsic and intrinsic apoptosis [115] pathways. Additionally, a recent study highlighted that circNUP50 contributes to platinum resistance in ovarian cancer by acting as a sponge for miR-197-3p and accelerating p53 ubiquitination, thereby reducing apoptosis and promoting resistance [116]. Studies have show that AKT signaling is crucial for various biological functions including cell proliferation and apoptosis. In MDR gastric cancer cells, estradiol cypionate reduces AKT protein expression by increasing ubiquitination, thereby inhibiting the overactivation of the PI3K-AKT-mTOR signaling pathway and suppressing cell growth [117]. In addition, AKT lysine-63 chain ubiquitination regulates AKT membrane localization and phosphorylation, which enhances the efficacy of cancer therapy [118].

### Ubiquitination and autophagy

Autophagy can degrade chemotherapeutic drugs, reduce the expression of drug targets, and remove drug damage to cancel cells, thus making them resistant to drugs [119]. Ubiquitination can affect autophagy by regulating the stability and activity of related proteins. For example, Linc00673-V3 inhibits the degradation of Smad3 through ubiquitination by the E3 ligase STUB1. Increased levels of Smad contribute to autophagy via LC3B transcription, leading to chemoresistance in NSCLC [120]. Additionally, miR-21-5p raises the level of LC3II/I by boosting the ubiquitination of Sirtuin7 (SIRT7) through ubiquitin-specific peptidase 42 (USP42), promoting deterioration and resistance to sorafenib in HCC cells [121]. Conversely, cancer cells may also use autophagy to degrade ubiquitin-labeled proteins, protecting them from intracellular proteasome degradation. This mechanism contributes to therapeutic resistance. Ubiquitin C-terminal hydrolase-L3 (UCH-L3) reduces β-catenin protein expression by inhibiting its ubiquitination during autophagy activation, aiding stress resistance in gastric cancer stem-like cells under nutrient deprivation [122]. Therefore, it is crucial to further investigate the link between ubiquitination and autophagy in cancer drug resistance.

### Ubiquitination and TME

The TME consists of a complex network of cells, blood vessels, ECM, and various molecules that surround tumor cells. TME significantly influences tumor resistance to therapy by affecting immune cell activation, ECM development, angiogenesis, and adaptation to both hypoxia and acidic environment [123, 124]. Hypoxia, a prevalent characteristic of the TME, significantly impacts tumor growth, metabolism, angiogenesis, and therapeutic resistance. It has been noted that the ubiquitination of hypoxia-inducible factor 1-alpha (HIF1α) is linked to resistance to the drug sorafenib. FASN interacts with HIF1α to promote its movement to the nucleus, thus preventing HIF1α's ubiquitination and degradation. This interaction enhances sorafenib-induced resistance to ferroptosis by boosting the expression of solute carrier family 7 member 11 (SLC7A11) [125]. Similarly, the ubiquitination of proteins such as steroid receptor coactivator 3 (SRC-3) and males absent on the first (MOF) has been shown to influence resistance associated with hypoxia in chemotherapy [126, 127]. Furthermore, ubiquitination also affects immunotherapy [128]. PD-L1, a critical immunomodulator that is regulated by ubiquitination. Tripartite motif 29 (TRIM29) triggers IGF2BP1 ubiquitination at Lys440 and Lys450 site via K48-mediated linkage, leading to protein degradation [129] and consequently enhancing PD-L1 mRNA stability and expression in 3′-UTR, which affects T-cell immunity in cancer. Additionally, E3 ligase speckle-type POZ protein (SPOP) mediates the ubiquitination and degradation of PD-L1, thus enhancing T cell–mediated cytotoxicity in HCC [130]. Cancer-associated fibroblasts (CAFs) are another key component in ECM formation [131]. Recent research has shown that reducing CD274 expression by removing K63-linked ubiquitination at the K280 residue can enhance the effectiveness of immunochemotherapy by suppressing CAF autophagy in PDAC [132]. These findings underscore the crucial role of ubiquitination in cancer therapy by influencing the TME.

### Ubiquitination and CSCs

Numerous studies have established that ubiquitination plays a critical role in the development of CSCs. For example, ubiquitination of β-catenin is associated with autophagy [122]. Additionally, β-catenin contributes to the properties of CSCs in lung adenocarcinoma (LUAD) [133]. The third-generation epidermal growth factor receptor–tyrosine kinase inhibitors such as osimertinib, have been used for clinical treatments. However, therapeutic failures are increasingly being reported [134]. Li et al. discovered that circFBXW7-185AA, a circular RNA from the gene F-box and WD repeat domain containing 7 (FBXW7), promoted the ubiquitination and subsequent instability of β-catenin. This action helps reverse resistance to osimertinib by reducing the activation of LUAD stem cells and affecting the Wnt signaling pathway function [133]. In addition, the E3 ubiquitin ligase FBXW7 interacts with the Notch 1 intracellular domain (NICD1), promoting NICD1 degradation through the ubiquitin–proteasome pathway. This process decreases the expression of stemness factors in CSCs and contributes to drug resistance in NSCLC [135]. Furthermore, Wang et al. identified that UBA1, an E1 ubiquitin-activating enzyme, triggers the degradation by regulating ubiquitination of RNA-binding-base-regulating protein 15 (RBM15), affects the therapeutic effectiveness of docetaxel in chemotherapy-resistant prostate cancer by influencing the stemness and apoptosis of prostate CSCs [136]. Understanding the intricate connection between ubiquitination and CSCs is crucial to overcoming cancer treatment resistance.

## Interplay between O-GlcNAcylation and ubiquitination in cancer therapeutic resistance

### O-GlcNAcylation regulated ubiquitination in cancer therapeutic resistance

**Fig. 5:**
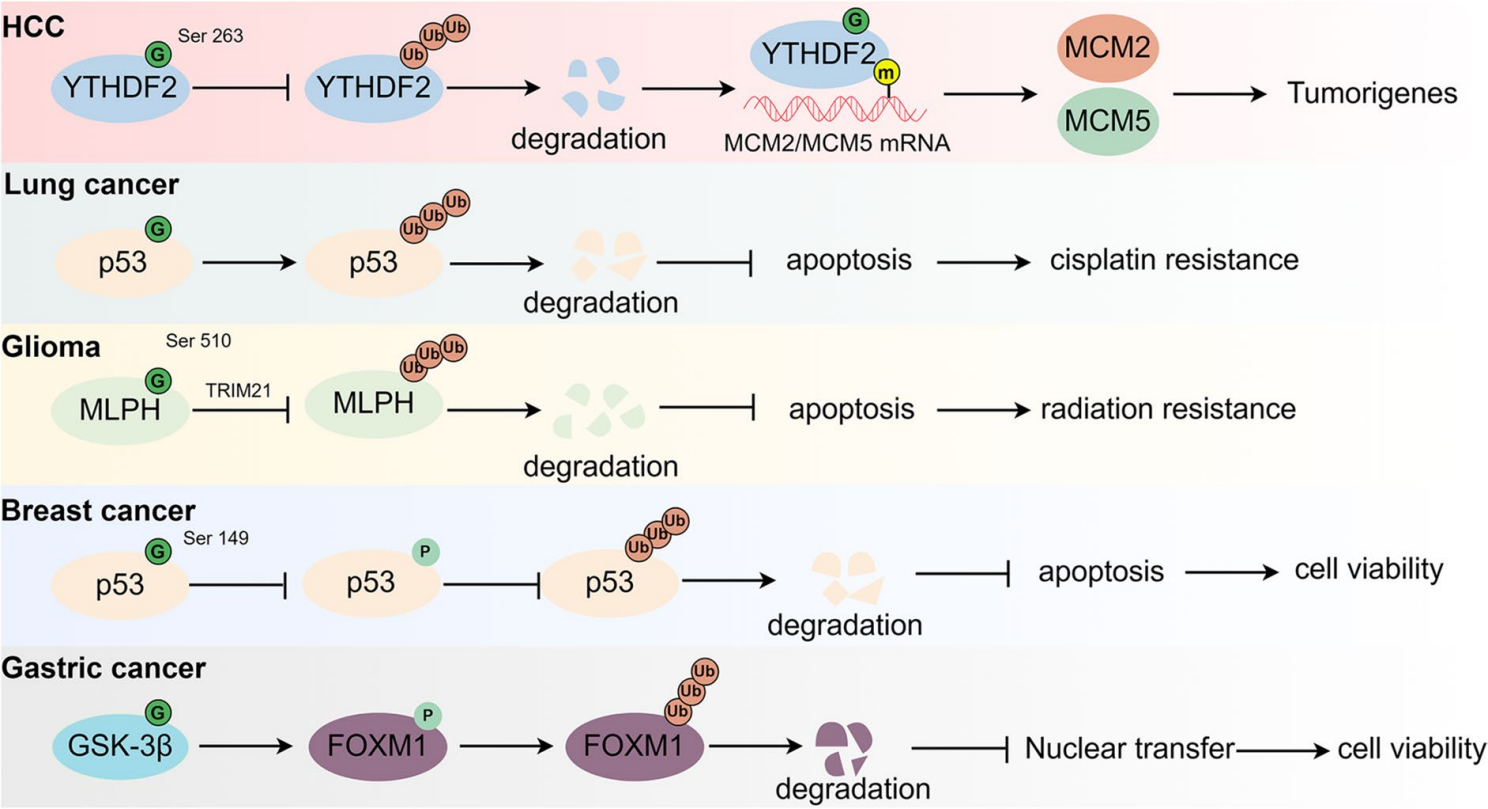
The role of O-GlcNAcylation in cancer therapeutic resistance. O-GlcNAcylation alter the structure and function of proteins, which may affect the recognition of ubiquitinating enzymes and the ubiquitination of substrates, thus regulating the roles of O-GlcNAcylation in therapeutic resistance of multiple cancers. (1) In HCC: the O-GlcNAcylation of YTHDF2 elevated its stability by inhibiting ubiquitination, thereby promoting MCM2 and MCM5 m6A modification, leading to tumorigenes. (2) In lung cancer: O-GlcNAcylation promoted p53 ubiquitin-mediated protein degradation, resulting in apoptosis and cisplatin resistance, respectively. (3) In Glioma: MLPH facilitated apoptosis and radiation resistance via decreasing MLPH ubiquitin-mediated protein degradation. (4) In breast cancer: O-GlcNAcylation of p53 facilitated its ubiquitin-mediated protein degradation through regulating its phosphorylation, thereby influencing apoptosis and cell viability. (5) In gastric cancer: O-GlcNAcylation of GSK-3β promoted the phosphorylation of FOXM1, and then contributing to its ubiquitin-mediated protein degradation, resulting in nuclear transfer and influencing cell viability

PTMs, particularly O-GlcNAcylation and ubiquitination, is associated with cancer therapeutic resistance. There is a certain interaction between these two pathways. Ubiquitination affects protein stability and localization, which may indirectly alter O-GlcNAcylation state. Conversely, O-GlcNAcylation may alter protein structure and function, potentially affecting ubiquitin enzyme recognition and substrate ubiquitination. These modifications might also compete for the same protein sites, influencing each other. This section provides a brief overview of current research on how O-GlcNAcylation affects ubiquitination in the resistance to treatments for cancers such as HCC, lung carcinoma, glioma, BC, and gastric cancer (Fig. 5).

#### Hepatocellular carcinoma

HCC is a type of malignant liver tumor usually associated with chronic liver inflammation or cirrhosis [137]. The most effective treatments are surgical resection or liver transplantation, but it frequently recurs and requires systemic treatment [138]. Recent research has highlighted the significant roles of O-GlcNAcylation and ubiquitination in HCC, particularly their interactions. For instance, Qiu et al. demonstrated that O-GlcNAcylation of the tumor suppressor p27 at Ser 2 increases the phosphorylation of p27 at Ser10, ultimately resulting in increased p27. This enhancement leads to more ubiquitination of p27, which promotes cell cycle progression and reduces interactions between the cyclin/CDK complex and p27 by encouraging its export of p27 in HCC from the nucleus [139]. Additionally, Yang et al. found that O-GlcNAcylation of YTHDF2 at Ser 263 by OGT increases its stability and cancer-promoting activity by reducing its ubiquitination. YTHDF2 stabilized the mRNA transcripts of minichromosome maintenance protein 2 (MCM2) and MCM5 in N6-methyladenosine (m6A)-dependent manner, thereby facilitating cell cycle progression and contributes to the development of HBV-related HCC (Fig. 5) [53]. Similarly, O-GlcNAcylation of YTHDF1 at Ser 197 promoted the binding of nuclear export signalling motifs to nucleocytoplasmic protein with exportin 1 (Crm1) by increasing hydrogen bonding [140]. While most studies indicate that O-GlcNAcylation generally stabilizes proteins by preventing their ubiquitin-mediated protein degradation, there are exceptions. Huang et al. reported that O-GlcNAcylation could enhance the ubiquitination and subsequent degradation of FOXA2in highly metastatic HCC cells, promoting O‐GlcNAcylation‐mediated HCC cell migration and invasion [58].

#### Lung cancer

Lung cancer is a major global health threat and a leading cause of cancer-related incidence and mortality worldwide. In 2018, approximately 2.09 million newly diagnosed cases of lung cancer were reported [141], representing 11.6% of all malignancies, and making lung cancer the most common malignancy. It also led to approximately 1.76 million deaths, which accounted for 18.4% of all cancer-related deaths [142]. Traditional treatments such as radiotherapy and chemotherapy usually have limited efficacy, especially for patients in intermediate and advanced stages of lung carcinoma, who generally experience high mortality rates and poor prognosis. Nonetheless, recent advancements in targeted therapies and immunotherapies have offered new hope. Despite these advances, many patients have developed resistance to these treatments over time. Therefore, it is crucial to understand the molecular mechanisms of acquired resistance in lung cancer.. Recent studies have emphasized the significant role of O-GlcNAcylation in influencing drug resistance in lung carcinoma. For example, hyper-O-GlcNAcylation could render lung carcinoma cells to apoptosis resistance through distinct mechanisms that involve p53 or c-Myc, depending on cellular context. high CDDP-induced p53 activation, hyper-O-GlcNAcylation targets p53, promotes its ubiquitination and subsequent p53 degradation, resulting in the gain of oncogenic and anti-apoptotic functions. By contrast with low p53 activation, hyper-O-GlcNAcylation has minimal effect on p53 and instead regulates c-Myc stability by interfering with its ubiquitin-mediated degradation. These notions are supported by the correlation analysis between O-GlcNAcylation and ubiquitination of p53 or c-Mycduring cisplatin treatment, affecting drug resistance (Fig. 5) [143]. Additional research has found that O-GlcNAcylation of caveolin-1 and c-Myc enhances protein stability by blocking their ubiquitination and proteasomal degradation. This reveals an important role of c-Myc O-GlcNAcylation in the motility of NSCLC [144].

#### Glioma

Gliomas are the most common type of malignant brain tumors, with glioblastoma multiforme being particularly aggressive and often fatal, resulting in a poor prognosis for patients [145, 146]. Although the exact mechanisms that cause gliomas to develop and progress remain unclear [146, 147], recent research has shed some light. Xu et al. reported that O-GlcNAcylation of melanophilin (MLPH) prevents its degradation by interacting with the E3 ubiquitin ligase tripartite motif containing 21 (TRIM21) (Fig. 5). This interaction appears to enhance glioblastoma's resistance to radiation by activating the NF-κB signaling pathway [148]. Another study highlighted the role of enhancer of zeste homolog 2 (EZH2) in controlling antitumor immunity in glioma. EZH2's structure is conducive to phosphorylation and O-GlcNAcylation, influencing glioma cell invasion and metastasis. Suppressing EZH2 expression and PTMs could potentially reverse resistance to temozolomide in patients with glioma [149].

#### Breast cancer

BC represents approximately 30% of all cancers in women globally, with a significant mortality-to-incidence ratio of 15%. With the annual increases in both incidence and mortality rates globally, identifying effective biomarkers for BC diagnosis and prognosis is crucial.Silent information regulator 1 (SIRT1), a NAD + -dependent deacetylase, plays significant roles in BC [150]. For example, Ferrer et al. confirmed SIRT1's critical role in the OGT-mediated regulation of Forkhead box M1 (FOXM1) ubiquitination, showing that reducing SIRT1 activity could mitigate OGT's effects on FOXM1, thereby influencing invasion and metastasis in BC cells [151]. Further research revealed that O-GlcNAcylation of ryanodine receptor 1 (RYR1) interferes with NEK10-mediated phosphorylation, increasing ubiquitination and proteasomal degradation; miR-122-mediated reductions in OGT led to higher RYR1 abundance in BC [152].In addition, Yang et al. found that O-GlcNAcylation of p53 at Ser 149 in MCF-7 cells reduced Thr 155 phosphorylation, rendering p53 partially resistant to ubiquitin-dependent proteolysis under streptozotocin treatment [153], affecting cell viability (Fig. 5). These findings underline the complex interactions between O-GlcNAcylation, phosphorylation, and ubiquitination in cancer therapy, highlighting the need for further study on the relationship between mutant proteins and O-GlcNAcylation, particularly p53 regulation and O-GlcNAcylation.

#### Gastric cancer

Gastric cancer is the second deadliest cancer worldwide, with approximately 60% of new cases of signet-ring cell carcinoma reported in East Asian countries [154, 155]. Understanding the molecular mechanisms that drive the progression and therapeutic resistance of GC is essential for improving diagnostic and treatment strategies. High glucose levels increase O-GlcNAcylation, which stabilizes the FOXM1 protein through reduced ubiquitin-mediated degradation due to GSK-3β inactivation in MKN45 cells. This process may increase the risk of gastric cancer in patients with diabetes (Fig. 5) [156]. However, studies on the roles of O-GlcNAcylation and ubiquitination in gastric cancer are limited, and more research is necessary to explore their functions in the disease's progression and resistance to treatment.

### Ubiquitination affected O-GlcNAcylation in *cancer* therapeutic resistance

O-GlcNAcylation plays a regulatory role in ubiquitination, which, in turn, can affect the level of O-GlcNAcylation in cancer. Ubiquitination primarily influences O-GlcNAcylation by regulating the stability of the enzyme OGT.

Increasing evidence from epigenetic studies indicates that aberrant epigenetic modifications, such as O-GlcNAcylation and ubiquitination, can contribute to the occurrence, progression, and therapeutic resistance in HCC. Notably, inhibition of USP8 can slow HCC progression and induce ferroptosis by reducing OGT stability through the inhibition of K48-specific poly-ubiquitination at the K117 site of the OGT protein in HCC cells. This finding highlights the potential of targeting USP8 as a therapeutic strategy for HCC [60]. Additionally, research has identified that the HECT-type E3 ubiquitin ligase E6AP targets OGT for ubiquitination and degradation, thereby affecting O-GlcNAcylation and various cellular processes in HEK293 cells [61]. This suggests that ubiquitination of OGT could be a novel therapeutic target for improving therapeutic outcomes in HCC treatment.

Osteosarcoma is a primary form of bone cancer that often develops during adolescence and is noted for its aggressive nature [157]. Despite improvements in surgical methods and chemotherapy, the overall 5-year survival rate for patients with osteosarcoma remains approximately 60% [158]. Therefore, understanding the molecular mechanisms driving the progression of osteosarcoma could reveal new therapeutic targets. Interestingly, Deng et al. suggested that suppressing Rho-associated coiled-coil forming protein kinase 2 (ROCK2) not only hinders osteosarcoma cell proliferation both in vivo and in vitro but also triggers apoptosis. ROCK2 also disrupts the tumor necrosis factor–related apoptosis-inducing ligand (TRAIL)-mediated apoptotic pathway, which enhances cell survival. Specifically, ROCK2 affects osteosarcoma progression and TRAIL resistance by modifying O-GlcNAcylation levels through the ubiquitin-mediated protein degradation of OGT. This highlights the critical role of OGT [159] ubiquitination in developing TRAIL resistance.

The mutual regulation of O-GlcNAcylation and ubiquitination in cancer therapy resistance and its influence on cancer therapy is a complex and important research area. Some mechanisms by which O-GlcNAcylation regulates ubiquitination are as follows. (1) Direct interaction: O-GlcNAcylation can occur on key proteins in the ubiquitination pathway, including E3 ubiquitin ligases, deubiquitinating enzymes (DUBs), and ubiquitin itself. This modification can directly affect the activity, stability or localization of these proteins, thereby regulating the ubiquitination process. (2) Affecting protein structure: The O-GlcNAcylation can alter the three-dimensional (3D) structure of a protein, which may affect the protein's binding to ubiquitin or other components of the ubiquitination pathway, which in turn affects the ubiquitination process. (3) Competitive modification: O-GlcNAcylation and ubiquitination may occur on the same amino acid residue, resulting in competitive inhibition between them. This competitive modification can regulate the ubiquitination state and subsequent degradation of the protein. (4) Regulation of enzyme activity: O-GlcNAcylation can regulate the activity of key enzymes in the ubiquitination pathway, such as E3 ubiquitin ligase and DUBs.

And ubiquitination can regulate O-GlcNAcylation mainly shows the function of regulating OGT and OGA, thereby affecting cancer therapeutics resistance. (1) Regulation of OGT and OGA: Ubiquitination can affect the stability and activity of OGT and OGA. For example, ubiquitination mediated degradation can reduce the level of OGT or OGA, thus affecting the overall level of O-GlcNAcylation. (2) Affecting protein interactions: ubiquitination can alter interactions between proteins, including those involved in the O-GlcNAcylation pathway. This may affect the binding of OGT and OGA to their substrates or regulators. (3) Affecting intracellular localization of proteins: ubiquitination can regulate the intracellular localization of proteins, including OGT and OGA. This alteration may affect their activity within cells or their access to substrates. The mutual regulatory effects of O-GlcNAcylation and ubiquitination in cancer therapy involve multiple levels, including regulation of protein stability and function, cross-regulation of signal transduction pathways, regulation of cell cycle and apoptosis, influence of DNA damage repair and genome stability, tumor microenvironment and immune response. Studying the interaction of these two modification pathways contributes to a deeper understanding of the mechanisms of cancer therapeutics resistance and provides new ideas for developing more effective anti-cancer strategies.

## Conclusions, challenges and prospects

Resistance to cancer treatments poses a significant challenge, often leading to increased mortality and poorer patient outcomes.Therefore, exploring the molecular basis of this resistance is crucial for improving treatment effectiveness. Various studies have identified multiple factors that influence tumor response to therapy; however, the PTMs involved in therapeutic resistance are not fully understood. This review presents recent findings on the interaction between O-GlcNAcylation and ubiquitination and its impact on resistance to cancer therapies. This review also explores potential strategies and future directions for overcoming such resistance.

Furthermore, PTMs, particularly O-GlcNAcylation and ubiquitination, are associated with several diseases, including cancer. The findings indicate that these modifications in tumor cells can lead to imbalances in processes such as the ABC transporter gene family activity, DNA damage and repair, autophagy, apoptosis, and TME. These imbalances contribute to cancer recurrence and resistance to therapy. Although it is well established that O-GlcNAcylation regulates ubiquitination in the context of cancer resistance, affecting protein degradation and localization, the research into how ubiquitination influences O-GlcNAcylation in response to cancer treatment is still in its early stages.

Identifying the interaction between O-GlcNAcylation and ubiquitination presents technical challenges. Recently, numerous technologies for detecting O-GlcNAcylation have been developed, including O-GlcNAc 4D-Label-free and LC–MS/MS analysis. O-GlcNAc 4D-Label-free, for example, combines ion mobility spectrometry with traditional mass spectrometry techniques [160, 161]. This method adds a third dimension of separation to high resolution and mass accuracy, enhancing peak capacity, separation quality, and quantitative accuracy. By applying this technique to quantitative proteomic studies of O-GlcNAcylation, researchers can better understand its distribution and role in various biological processes. This advancement is crucial for exploring the dynamics of O-GlcNAcylation in health and disease and improving the detection of O-GlcNAcylation and ubiquitination.

In recent studies, numerous natural products and lead compounds targeting O-GlcNAcylation have been identified. For instance, Lee et al. reported that the combined treatment of HCT116 cells with metformin and OSMI-1, an O-GlcNAcylation inhibitor, significantly increased apoptosis by enhancing ER stress-induced pathway signaling, rather than promoting protective autophagy in colon cancer treatment [162]. Furthermore, Zhu et al. demonstrated that introducing a short peptide sequence containing O-GlcNAcylation sites of the HGS into cells could competitively inhibit HGS O-GlcNAcylation with minimal overall effect on cellular O-GlcNAcylation levels. This approach led to decreased PD-L1 expression and increased T cell cytotoxicity [95], indicating that specific proteins with unique site-specific O-GlcNAcylation play crucial roles in cancer development and progression.

In recent years, ubiquitination has emerged as a significant focus for cancer therapy. However, its role in cancer treatment resistance is complex, marked by both potential and limitations [163]. A primary obstacle is the broad functions of ubiquitin ligases and deubiquitinating enzymes (DUBs) [164]. These enzymes interact with numerous substrates across various signaling pathways, complicating the targeting of specific cancer-related ubiquitination events without unintended consequences. Moreover, in cancer cells, altered ubiquitination can contribute to resistance by either degrading tumor suppressors and anticancer drugs or by stabilizing oncogenes and cell survival proteins [107, 108]. Despite these difficulties, promising avenues exist for developing ubiquitination-based cancer therapies. Advances in proteomics and ubiquitomics have enabled the identification of precise ubiquitination targets within cancer cells, potentially restoring therapy sensitivity. Furthermore, research into small-molecule inhibitors that specifically target ubiquitin ligases or DUBs is active and might enhance the effectiveness of existing cancer treatments by overcoming resistance [165]. In summary, although challenges remain for cancer therapies targeting uniquitination, continued research and technological progress hold promise for innovative strategies to overcome treatment resistance and improve patient outcomes.

In conclusion, the interaction between O-GlcNAcylation and ubiquitination is crucial in managing tumor therapeutic resistance. O-GlcNAcylation influences the stability, activity, and localization of proteins vital for cell cycle regulation, DNA damage repair, and apoptosis—all essential in cancer progression and response to therapy. Conversely, ubiquitination tags proteins for degradation, influencing both O-GlcNAcylation proteins and other critical signaling proteins for cancer cell survival and death. The balance between these two PTMs is delicate and is often disrupted in cancer, promoting therapeutic resistance. Studies primarily investigate the role of GlcNAcylation in protein stability and function through its effect on ubiquitination, while ubiquitination can affect O-GlcNAcylation by altering OGT and OGA levels. Targeting the enzymes linked to O-GlcNAcylation and ubiquitination, such as OGT and ubiquitin-specific proteases, respectively, may help overcome resistance to cancer therapies. Additionally, elucidating the interactions between these two pathways could lead to the development of novel therapeutic approaches that disrupt cancer cells' resistance mechanisms.

## Data Availability

No datasets were generated or analysed during the current study.

## Abbreviations

8-Mar: Membrane-associated RING-CH 8
ABCB1: ABC subfamily B member 1
ATP: Adenosine triphosphate
BCL-2: B-cell lymphoma 2
BetA: Betulinic acid
BTZ: Bortezomib
CAFs: Cancer-associated fibroblasts
Cat B: Cathepsin B
CRPC: Chemotherapy resistance prostate cancer
CSCs: Cancer stem cells
CSCs: Cancer stem cells
CTGF: Connective tissue growth factor
DDR: DNA damage response
DNMT1: DNA methyltransferase 1
DSBs: DNA double strand breaks
DUBs: Deubiquitinating enzymes
E1: Ubiquitin-activating enzyme
E2: Ubiquitin-binding enzyme
E3: Ubiquitin ligase
ECM: Extracellular matrix
ECP: Estradiol cypionate
EOGT: EGF domain-specific O-GlcNAc transferase
EZH2: Enhancer of zeste homolog 2
F-6-P: Fructose-6-p
FASN: Fatty acid synthase
FASN: Fatty acid synthase
FBXW7: F-box and WD repeat domain containing 7
FOXA2: Factor forkhead box protein A2
FOXA2: Forkhead box protein A2
FOXM1: Forkhead box M1
GB: Glioblastoma
GBM: Glioblastoma multiforme
GC: Gastric cancer
GFAT/GFPT: Glutamine-fructose-6-phosphate aminotransferase
GFAT1: Glutamine-fructose-6-phosphate aminotransferase (isomerizing) 1
HBP: Hexosamine biosynthetic pathway
HBV: Hepatitis B virus
HCC: Hepatocellular carcinoma
HCC: Hepatocellular carcinoma
HGS: Hepatocyte growth factor-regulated tyrosine kinase substrate
HGS: Hepatocyte growth factor-regulated tyrosine kinase substrate
HIF1α: Hypoxia-inducible factor 1-alpha
HRR: Homologous recombination repair
IMS: Ion mobility spectrometry
IR: Irradiation
LUAD: Lung adenocarcinoma
m6A: N6-methyladenosine
MCM2: Minichromosome maintenance protein 2
MCM5: Minichromosome maintenance deficient 5
MDR: ABCB1-mediated multidrug resistance
MGEA5: Meningioma-expressed antigen-5
MLPH: Melanophilin
MOF: Males absent on the first
MRE11: Meiotic recombination 11 homolog 1
MTHFD2: Methylenetetrahydrofolate dehydrogenase 2
NICD1: Notch 1 intracellular domain
Nrf1: Nuclear factor erythroid 2-related factor
NSCC: Non-small cell cancer
NSCLC: Non-small cell lung cancer
OC: Ovarian cancer
OGA: O-GlcNAcase
OGT: O-GlcNAc transferase
PC: Prostate cancer
PCK1: Phosphoenolpyruvate carboxykinase 1
PDAC: Pancreatic ductal adenocarcinoma
PDAC: Pancreatic ductal adenocarcinoma
PD-L1: Programmed-death ligand 1
PMDs: Partially methylated domains
Polη: Polymerase η
PTM: Post-translational modification
pVHL: Von Hippel-Lindau tumor suppressor protein
RBM15: RNA Binding Motif Protein 15
RNF126: Ring finger protein 126
ROCK2: Rho-associated coiled-coil forming protein kinase 2
RYR1: Ryanodine receptor 1
SF3A2: Splicing factor 3a subunit 2
SIRT1: Silence information regulator 1
SIRT7: Sirtuin7
SKP2: S-phase kinase-associated protein 2
SLC7A11: Solute carrier family 7, member 11
SLC7A11: Solute carrier family 7 member 11
SM15: Small molecule 15
SNAIL: Snail family transcriptional repressor 1
SNAP-29: Synaptosomal-associated protein 29
SNARE: Soluble NSF attachment protein receptor
SPOP: Speckle-type POZ protein
SRC-3: Steroid receptor coactivator 3
STZ: Streptozotocin
TAMs: Tumor-associated macrophages
TCA: Tricarboxylic acid
TGF-β1: Transforming growth factor-β1
TKIs: EGFR- Tyrosine kinase inhibitors
TLS: Translesion DNA synthesis
TMDs: Typically comprises transmembrane domains
TME: Tumor microenvironment
TMZ: Temozolomide
TNBC: Triple-negative breast cancer
TRAIL: Tumor necrosis factor-related apoptosis-inducing ligand
TRIM21: Tripartite motif containing 21
TRIM29: Tripartite motif 29
Ub: Ubiquitin
UCH-L3: Ubiquitin C-terminal hydrolase-L3
UDP-GlcNAc: Uridine diphosphate N-acetylglucosamine
USP42: Ubiquitin-specific peptidase 42
USP8: Ubiquitin specific peptidase 8
YAP: Yes-associated protein
